# An update on the LIM and SH3 domain protein 1 (LASP1): a versatile structural, signaling, and biomarker protein

**DOI:** 10.18632/oncotarget.3083

**Published:** 2014-12-31

**Authors:** Martin F. Orth, Alex Cazes, Elke Butt, Thomas G. P. Grunewald

**Affiliations:** ^1^ Institute for Clinical Biochemistry and Pathobiochemistry, University Clinic of Würzburg, Grombühlstrasse, Würzburg, Germany; ^2^ Laboratory for Pediatric Sarcoma Biology, Institute of Pathology of the LMU Munich, Thalkirchner Strasse, Munich, Germany

## Abstract

The gene encoding the LIM and SH3 domain protein (LASP1) was cloned two decades ago from a cDNA library of breast cancer metastases. As the first protein of a class comprising one N-terminal LIM and one C-terminal SH3 domain, LASP1 founded a new LIM-protein subfamily of the nebulin group. Since its discovery LASP1 proved to be an extremely versatile protein because of its exceptional structure allowing interaction with various binding partners, its ubiquitous expression in normal tissues, albeit with distinct expression patterns, and its ability to transmit signals from the cytoplasm into the nucleus. As a result, LASP1 plays key roles in cell structure, physiological processes, and cell signaling. Furthermore, LASP1 overexpression contributes to cancer aggressiveness hinting to a potential value of LASP1 as a cancer biomarker.

In this review we summarize published data on structure, regulation, function, and expression pattern of LASP1, with a focus on its role in human cancer and as a biomarker protein. In addition, we provide a comprehensive transcriptome analysis of published microarrays (n=2,780) that illustrates the expression profile of LASP1 in normal tissues and its overexpression in a broad range of human cancer entities.

## 1. Domain organization and structure of human LASP1

**Figure 1:**
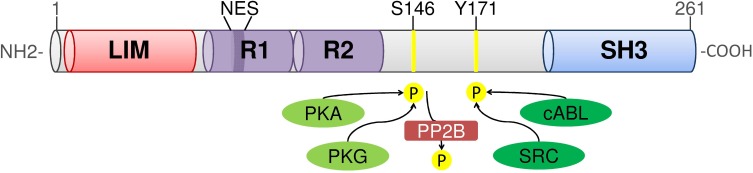
Domain organization of human LASP1 Functional domains and phosphorylation sites are shown. NES: nuclear export signal; PKA: cAMP-dependent protein kinase; PKG: cGMP-dependent protein kinase; PP2B: protein phosphatase 2B.

In 1995 Tomasetto *et al.* identified four genes in a cDNA library of metastatic axillary lymph nodes from breast cancer, one of them was termed *MLN50* [1]. *MLN50* was found to be located on chromosome 17q11-21.3. The transcribed 4.0kb mRNA encodes a protein of 261 amino acids (Fig. 1). This protein contains an N-terminal LIM domain, which is composed of two sequential zinc-binding modules with a typical LIM motif, followed by tandem 35-residue nebulin-like repeats named R1 and R2, and by a C-terminal SRC homology region 3 (SH3) domain. Therefore MLN50 was renamed LIM and SH3 domain protein 1 (LASP1) [2]. As LASP1 is the first protein that combines both, LIM and SH3 domains, and as it contains two nebulin-like repeats, it defines a new LIM protein subfamily of the nebulin group [2,3].

This unique domain composition renders LASP1 to be a potentially extremely versatile protein and may shed light on its diverse cellular functions: The conserved zinc-binding modules are structurally related to the DNA-binding zinc-finger domains of nuclear hormone receptors and likely form functionally independent folding-units, suggesting that LIM domains may bind to DNA [4]. The R1 and R2 repeats serve as interfaces for multiple protein-protein interactions and are often observed in scaffolds and stabilizers of the cytoskeleton [5]. The SH3 domain is a 60 amino acids segment shared by diverse structural and signaling proteins [6], first identified as a conserved sequence of the SRC protein tyrosine kinase [7].

The second protein of the LASP-subfamily, LASP2, was identified by bioinformatic prediction [8]. LASP2 also contains one LIM and SH3 domain, but differs from LASP1 in the number of nebulin-like repeats (3 versus 2). However, little is known about its expression patterns and molecular functions (for review [9]).

To date, numerous binding partners of LASP1 have been identified (Table 1). The LIM domain is hypothesized to be involved in homodimerization [4]. However, no LASP1 dimer or polymers have been reported so far *in vivo* [10]. The only known binding partner of the LASP1 LIM domain is the chemokine receptor 2 (CXCR2), which plays an important role during migration of epithelial and immune cells, in angiogenesis, inflammation, wound healing, and atherosclerosis. Site-directed mutagenesis identified direct binding of the LASP1 LIM domain with the C-terminal domain of CXCR2. For CXCR1, CXCR3, and CXCR4 binding to LASP1 has been shown as well. As many residues in the C-terminal domains of CXCRs are conserved, the binding may also occur at the LIM domain of LASP1. However, this has not been proven in detail so far [11]. Indeed, the interaction between LASP1 and CXCR2 is critical for CXCR2-mediated chemotaxis, suggesting a coordinating role of LASP1 between the CXCRs and nascent focal adhesions [11].

**Table 1:** Overview on the LASP1 protein domains, the LASP1 binding partners and their corresponding binding sites, as well as putative functions

LASP1 domain	Binding partner			References
	Name	Identified binding site	Main functions	
LIM	CXCR2	C-terminal domain (Iso323-Leu324)	G protein-linked receptor involved in migration of immune and endothelial cells, modulating inflammation and atherosclerosis	[11]
R1/R2	F-actin	-	Major component of the cytoskeleton, necessary for cell movement, intracellular signaling and trafficking	[12]
	Krp1	first and last of five Kelch repeats, that are in close proximity by formation of a six-bladed β-propeller structure	Cytoskeleton-interaction protein, plays role in elongation of pseudopodia and myofibril assembly	[13,14]
SH3	dynamin	proline-rich domain	GTPase, regulates fission of vesicles from the plasma membrane	[19,20]
	LPP	proline-rich pre-LIM region	Cytoskeletal and signaling protein, shuttles into the nucleus	[10,16,20]
	palladin	proline-rich motif at the N-terminus	Cytoskeletal scaffold, organization of the actin cytoskeleton	[18]
	VASP	proline-rich region in the mid-region	Actin binding partner, links signaling pathways to actin cytoskeleton remodeling	[10,17]
	ZO-2	within first proline-rich motif at the C-terminus (amino acids 1103-1109)	Regulates tight junctions, nuclear transport and binds to DNA-scaffolding and transcription factors	[20]
	zyxin	extreme N-terminus (amino acids 31-39)	Organization of the actin cytoskeleton, focal adhesions	[15]

The nebulin-like repeats mediate the interaction of LASP1 and filamentous actin (F-actin) [12] as well as binding to Kelch-related protein 1 (Krp1), which is involved in pseudopodial elongation [13,14].

Via its SH3 domain, LASP1 directly binds to proline-rich domains of several other proteins, namely zyxin [15], the LIM domain containing preferred translocation partner in lipoma (LPP; a shuttle protein and transcription factor) [16], vasodilator-stimulated phosphoprotein (VASP, an actin binding partner) [10,17], palladin (140kDa isoform) [18], dynamin [19], and zonula occludens protein 2 (ZO-2) [20], with zyxin being the most prominent interaction partner. Zyxin is, like LPP, a nucleo-cytoplasmic shuttle protein and acts as a versatile scaffold of focal adhesions in eukaryotic cells, regulating cellular movement as well as gene transcription within the nucleus [21,22]. LASP1 binding to zyxin occurs at the extreme N-terminus [15].

In addition, LASP1 contains two well-characterized phosphorylation sites: Serine 146 is a specific phosphorylation site for cGMP-(PKG) and cAMP-dependent protein kinases (PKA) [23], and is dephosphorylated by phosphatase PP2B [20]. Tyrosine 171 is the phosphorylation site for SRC- and ABL-kinase [12,24,25]. Phosphorylation of LASP1 at serine 146 modulates the interaction with several binding partners as for instance it decreases binding to actin, zyxin and LPP [20,23]. This phosphorylation-dependent modulation of protein-protein interaction allows a subcellular relocalization of LASP1 between sites of focal adhesions and the nucleus, which will be discussed in further detail below [20,23].

## 2. Expression patterns of LASP1 in normal tissues

**Figure 2:**
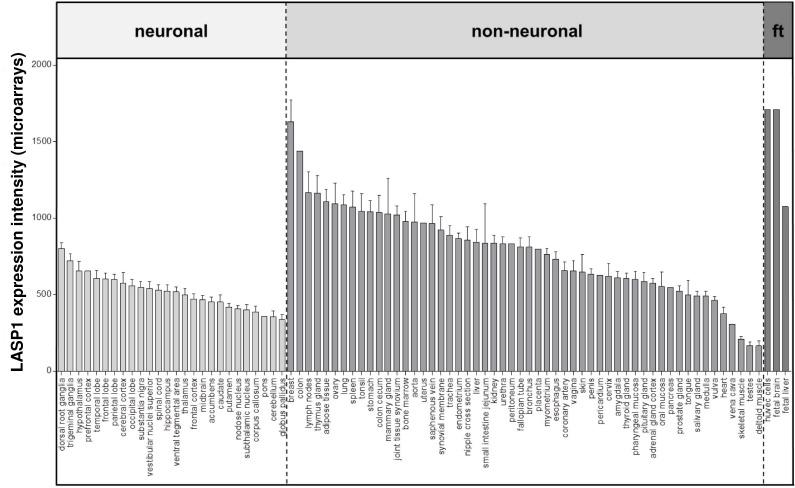
LASP1 is expressed at very distinct levels within normal tissues Publicly available microarray datasets (all Affymetrix HG-U133plus2.0) for n=502 normal tissues (GSE7307) were retrieved from the Gene Expression Omnibus (GEO) and normalized simultaneously by Robust Multi-array Average (RMA) using customized brainarray CDF files (ENTREZG v17) yielding one optimized probe-set for each transcript [106, 107, 108, 109]. Expression values are presented in bar plots as mean relative expression and standard error of the mean in descending order for neuronal, non-neuronal and fetal tissues (ft).

LASP1 is ubiquitously expressed in normal tissues, albeit at very different levels. LASP1 expression appears to be enriched in certain actin-rich cell and tissue types such as gastric parietal cells [26]. Here we present a comprehensive analysis of publicly available gene expression microarray data, which revealed up to 10-fold differences of LASP1 expression levels in non-neuronal tissues, while its expression levels in various neuronal tissues appears rather similar (Fig.2).

## 3. Structural and functional similarities and differences across species

Murine *Lasp1* is highly similar to its human counterpart. The gene is located at 11C-11D region on chromosome 11. Expression occurs from 7.5 to 17.5 days post coitum of embryogenesis and is detectable in almost all adult tissues [27]. Insertion of a transgene in this genomic region causes skeletal malformation [28], albeit without gene interruption. But as murine *Lasp1* is close to the locus of the transgene an interaction was assumed, suggesting a crucial role of the protein in early development and osteogenesis.

Exposure of murine cells to forskolin and concomitant activation of PKA induces a phosphorylation at threonine 156 (the corresponding murine amino acid of the human serine 146) and translocation of mouse LASP1 from focal contacts to the cytoplasm without affecting F-actin structure. Thus, although the human and murine PKA phosphorylation sites are different, there seems to be no functional difference [10].

Rabbit LASP1 exhibits 93.5% homology to human LASP1 [29]. PKA phosphorylation sites of the rabbit homologue are located at serine 99 and serine 146, respectively [30].

But expression of LASP1 homologues is not restricted to vertebrates: Genes encoding proteins homologous to LASP1 with partially conserved exon-intron boundaries were deposited in the genome/cDNA database of invertebrates such as sea urchins, nematodes and insects. Moreover, in *Ciona intestinalis* LASP1 showed actin-binding activity and was apparent in the nervous system of early embryos suggesting an analog function of invertebrate LASP1 compared to vertebrate or rather human LASP1 [31]. Yet, there are differences in the structure of insect LASP1 and the vertebrate proteins such as a longer sequence between the LIM and the SH3 domain [32].

In Drosphila, the LASP1 homologue is the only representative of the nebulin-family. The protein is localized at the Z-disc edges to control the architecture of I-band and A-band in sarcomeres, interacting both with actin and myosin setting proper filament spacing [33]. This is consistent with previous findings of human LASP1 at A-bands and Z-discs [3]. Moreover, in Drosophila, LASP1 homologue is essential for development of functioning reproductive organs: In Drosophila females, the protein binds and anchors Oskar protein to actin at the posterior pole of the oocyte, which is essential for induction of germ-plasm assembly [34]. The LASP1 homologue interacts with betaPS integrin and cooperates with integrins in hub cells to anchor the stem cell niche [35]. In male Drosophila, LASP1 homologue null mutants are sterile because of improper spermatid individualization. LASP1 homologue knockout disrupts actin cone migration, thus the stem cell niche is no longer anchored to testis [35].

## 4. LASP1 interaction with the cytoskeleton

Initial studies demonstrated a colocalization of LASP1 with sites of dynamic F-actin assembly and led to the identification of the functional actin-binding nebulin-like repeats in the core of the protein [12]. In contrast, binding of LASP1 to elongated actin stress fibers is indirect and mediated via interaction with the binding partner palladin [18].

LASP1 accumulates in focal adhesions, as well as in the leading edges of lamellipodia and tips of filopodia - the key structures for enabling cell adhesion, migration, and cellular communication [30,36]. This might be mediated by its SH3 domain as this domain enables LASP1 to bind to proline-rich domains of cytoskeleton proteins like zyxin, VASP and LPP - a common protein-protein interaction for proteins located close to the plasma membrane [7].

Photobleaching experiments with GFP-tagged actin and LASP1 transfected neuronal cells elucidated the complex actin filament organization: F-actin recovered inwards, whereas LASP1 recovery occurs from the anterograde direction, suggesting a participation of LASP1 in the stabilization, but not in the initiation of actin bundles [37]. Binding of LASP1 to F-actin is phosphorylation-dependent, as phosphorylation by PKG and PKA at serine 146 reduces its affinity to F-actin, albeit without affecting F-actin structure, and induces LASP1 translocation from focal contacts to the cell interior [23]. Therefore, LASP1 appears to be involved in cytoskeletal organization and cell motility [10,23].

Furthermore the translocation of LASP1 to F-actin-rich lamellipodial extensions and nascent focal complexes correlates with protein kinase C activation via PMA in gastric fibroblasts. As LASP1 is not directly phosphorylated by protein kinase C, an indirect mechanism for LASP1 translocation is assumed [30].

Phosphorylation of LASP1 at tyrosine 171 by SRC- and ABL-kinase does not affect the dynamics of migratory processes. Although activation of ABL-kinase by apoptotic agents leads to loss of LASP1 localization to focal adhesions and even to cell death, phosphorylation does not inhibit LASP1 localization to F-actin rich structures [24]. In contrast, upon fibrinogen binding integrin-activated phosphorylation of LASP1 by SRC-kinase in platelets results in LASP1 translocation to focal contacts and membrane ruffles which is associated with increased cell spreading [25].

Interestingly, LASP1 and LASP2 appear to have counteracting roles at focal adhesions: The integral focal adhesion proteins vinculin and paxillin are binding partners of LASP2, which in turn enhances their interaction. This interaction can be impaired by LASP1. Accordingly, the dynamic interplay of LASP1 and LASP2 at focal adhesions could mechanistically control cell adhesion by regulation of focal adhesion assembly [36].

LASP1 is also a component and regulator of pseudopodia and podosomes. Translocation of LASP1 to the leading edge of pseudopodia from the periphery is increased by stimulation with growth factors or extracellular matrix proteins in an c-Abl kinase activity-dependent but tyrosine phosphorylation independent manner [24]. At the tips of pseudopodia, LASP1 interacts with Krp1 and appears to be necessary for pseudopodial extension and invasion. Although the SH3 domain of LASP1 is not involved in Krp1-binding, deletion of this domain led to truncated pseudopodia and less invasiveness, proving the importance of the SH3 domain for pseudopodial formation, extension, and invasion [13,14].

Podosomes are highly dynamic structures that are actively engaged in matrix remodeling and degradation. They are composed of an actin-rich core region surrounded by a ring-like structure containing signaling molecules, motor proteins, and cytoskeleton-associated proteins including LASP1. LASP1 is already recruited in an early step of podosome assembly and colocalizes with zyxin and vinculin, suggesting an initiator function. Consistently, siRNA-mediated knockdown of LASP1 negatively affects podosome dynamics and matrix degradation [38].

Though, LASP1 is necessary for cell migration and cellular survival, surprisingly all studies failed to show an impairment of adhesion upon LASP1 knockdown [24].

LASP1 and its interaction with the cytoskeleton can also be modified by mechanisms other than phosphorylation as the protein shows increased tyrosine nitration in an age- and systemic inflammatory response syndrome-dependent manner in mice, concomitant with protein tyrosine nitration of other components of the actin cytoskeleton [39]. However, the relevance of this modification is still unknown.

## 5. LASP1 in physiological processes

The interaction of LASP1 with various binding partners via its unique domain organization and its ability to translocate between the cytoplasm, focal contacts, cell extensions and nucleus exhibits not only structural, but also functional and regulatory significance and has been investigated in several model systems:

### 5.1. Role of LASP1 in physiological function of gastric parietal cells

In 1998, Chew and co-workers reported a secretory HCl response in correlation to LASP1 phosphorylation in forskolin stimulated rabbit gastric parietal cells [29]. Similar observations were made later on for the corresponding human cells, which express LASP1 at a high level. Physiologically, histamine stimulates PKA mediated LASP1 phosphorylation in parietal cells, which is closely correlated with the acid secretory response and induces a relocation of the protein in these cells: LASP1 is mainly localized at the cell cortex along with the γ-isoform of actin in non-stimulated cells, upon phosphorylation it localizes to the apical β-actin enriched intracellular canalicular region, the site of active proton transport [26]. Therefore substitution of PKA phosphorylation sites serine 99 and serine 146 in parietal cells of rabbits suppressed cAMP-dependent translocation of LASP1 to the intracellular canalicular region [30]. Consistently, in cholera toxin transgenic mice, the constantly increased cAMP levels led to hyperphosphorylation of LASP1 and consequently to elevated gastric acid content, which was accompanied by reduced levels of circulating stimulators of acid secretion such as gastrin [40]. Gastrin itself appeared to have an exclusive effect on LASP1 phosphorylation, as in gastrin-deficient mice a marked reduction in LASP1 phosphorylation without affecting mRNA or protein levels in parietal cells was observed [41].

Surprisingly, in LASP1 knockout mice the basal HCl secretion was unaffected, but histamine stimulation induced a more robust acid secretory response. Conversely, inhibition of the histamine response by H2 receptor blockade with ranitidine was delayed, pointing to a regulatory function of LASP1 in parietal HCl secretion [42]. Accordingly it is tempting to assume, that PKA mediated LASP1 phosphorylation affects interaction with F-actin and endocytic proteins, thus modulating both, trafficking and activation, of the H+/K+-ATPase [42]. Herein, dynamin is a likely mediator, which may bind to F-actin binding LASP1, thereby linking the vesicular trafficking machinery with the cytoskeleton [19].

### 5.2. Role of LASP1 in renal function

Vesicular trafficking regulates the activity of several apical membrane transporters. The gastric HCl-secreting parietal cell is only one model for this system [19]. Another example is the kidney collecting duct cell, likewise prominently expressing LASP1 [26]: Here, aquaporin-2 trafficking to the apical plasma membrane is driven by vasopressin-induced apical F-actin dynamics. LASP1 was identified as a protein being significantly and strongly modulated in its abundance in the apical plasma membrane of murine duct cells in response to vasopressin, suggesting a role in the aquaporin-2 trafficking [43].

### 5.3. LASP1 in neural cells

LASP1 is strongly expressed in CNS neurons and a role in several neurologic and psychiatric disorders is discussed.

High LASP1 expression levels were found in cortex, cerebellum, and hippocampus and LASP1 is concentrated at synaptic sites as one out of many actin-associated proteins in postsynaptic density fractions. This suggests many modes of action by which the state of synaptic F-actin polymerization and hence, synaptic physiology can be affected [44]. In an organelle proteomic approach for rat synaptic proteins, LASP1 was confirmed to be enriched in the postsynaptic density fractions, but was also present in other subdomains of the synapse [45]. During neuronal differentiation of hippocampal neurons, LASP1 is first observed in growth cones and later begins to distribute throughout the dendrites with subsequent clustering at postsynaptic densities of dendritic spines [44]. Indeed, LASP1 expression is regulated in hippocampus during postnatal brain development as seen for Sprague-Dawley rats [46] and is upregulated in response to nerve growth factor β-NGF [47]. Collectively, these data hint to a possible involvement of LASP1 in neuronal development. In line with this hypothesis are four lines of evidence made in psychiatric disorders:

First: LASP1 is downregulated in mice treated with MK-801, the non-competitive antagonist of the NMDA receptor that constitutes an important animal model for schizophrenia studies. This association of LASP1 downregulation with schizophrenia in a mouse model is supported by a single nucleotide polymorphism (SNP) in the human *LASP1* gene promoter region on chromosome 17, known to be associated with the susceptibility for schizophrenia in Korean population [48].

Second: Affected sibling pair linkage analysis identified a region on chromosome 17 that has been linked to autism, with an even stronger linkage in families with only males affected. A high density SNP study in this region detected a significant association of a SNP in the *LASP1* gene to autism. But this association was not sufficient to account for the initial linkage signal of the region on chromosome 17 [49].

Third: A comparative proteomic analysis of peripheral lymphocytes from patients affected by acute psychotic bipolar disorder or by major depressive episodes with no personal or family history of psychosis identified 25 differentially expressed proteins in patients compared to matched healthy controls. LASP1 is one out of the two proteins being significantly upregulated in patients with psychotic bipolar disorder. These findings suggest an additional LASP1-dependent mechanism associated with the psychotic features of bipolar disorders [50].

Fourth: LASP1 is significantly upregulated in the hippocampus of rats exposed to early life stress, the standard model in studies of epigenetic programming that leads to mood disorders and anxiety in adult age [51].

### 5.4. Role of LASP1 in chondro- and osteogenesis

To date, several studies link LASP1 expression levels to chondrogenic and osteogenic differentiation processes, yet with still unclear spatiotemporal and mechanistic resolution.

For instance, LASP1 is increased in beagle bone marrow stem cells by human bone morphogenetic protein-2 during induction of osteogenic differentiation [52]. In contrast, LASP1 is also upregulated in later passages of human mesenchymal stem cells with a decreased potential of osteogenic differentiation [53].

Evidence for the role of LASP1 in chondrocyte differentiation during endochondral ossification has been obtained in the mouse mutant wavy tail Tg(Col1a1-lacZ)304ng. In this mutant, transgene insertion near the transcription start site of the *Lasp1* gene causes a temporally and spatially LASP1 misexpression in the vertebra. The mice exhibit defects of the vertebral column, delayed closure of lumbar neural arches and lack of processus spinosi, which become most prominent during the transition from cartilage to bone. [54].

Therefore, LASP1 seems to be linked with both intramembranous and endochondral ossification. This notion gains support by the fact that LASP1 is downregulated in osteoarthritis chondrocytes upon stimulation with the proinflammatory cytokine IL-1β, that induces changes in the morphology of chondrocytes and the organization of the cytoskeleton [55].

### 5.5. LASP1 as a possible mediator of the effect of homocysteine?

Hyperhomocysteinemia is a common independent risk factor for cardiovascular diseases. The promoting effect of homocysteine on vascular smooth muscle cell proliferation has been considered as one of the important pathological bases of atherosclerosis. Comparing the protein expression profiles of homocysteine treated and non-treated vascular smooth muscle cells, revealed an alteration of 11 proteins, thereof LASP1 being downregulated markedly [56]. Hyperhomocysteinemia is also a risk factor for neuronal lesions and is often accompanied with high levels of homocysteic acid (HCA). In this context, HCA induced LASP1 tyrosine phosphorylation [57].

### 5.6. LASP1 - a regulator of insulin sensitivity?

In one study, testing the insulin sensitizer rosiglitazone in obese, the investigators observed major changes in abdominal subcutaneous adipose tissue among proteins involved in insulin and calcium regulation, inflammatory and redox signals, as well as glucose-transporter-4 granule transport and fusion. Regulated proteins involved in cytoskeleton rearrangement were identified as actin, myosin-9, annexins, vimentin, tubulin, and LASP1. However no changes in mRNA expression were detected, suggesting adaptation at a post-transcriptional level in response to rosiglitazone [58]. Accordingly, a role of LASP1 in regulation of glucose transporter-4 expression and insulin sensitivity can be considered, but the mechanisms are unknown so far.

## 6. LASP1 in human cancer

**Figure 3:**
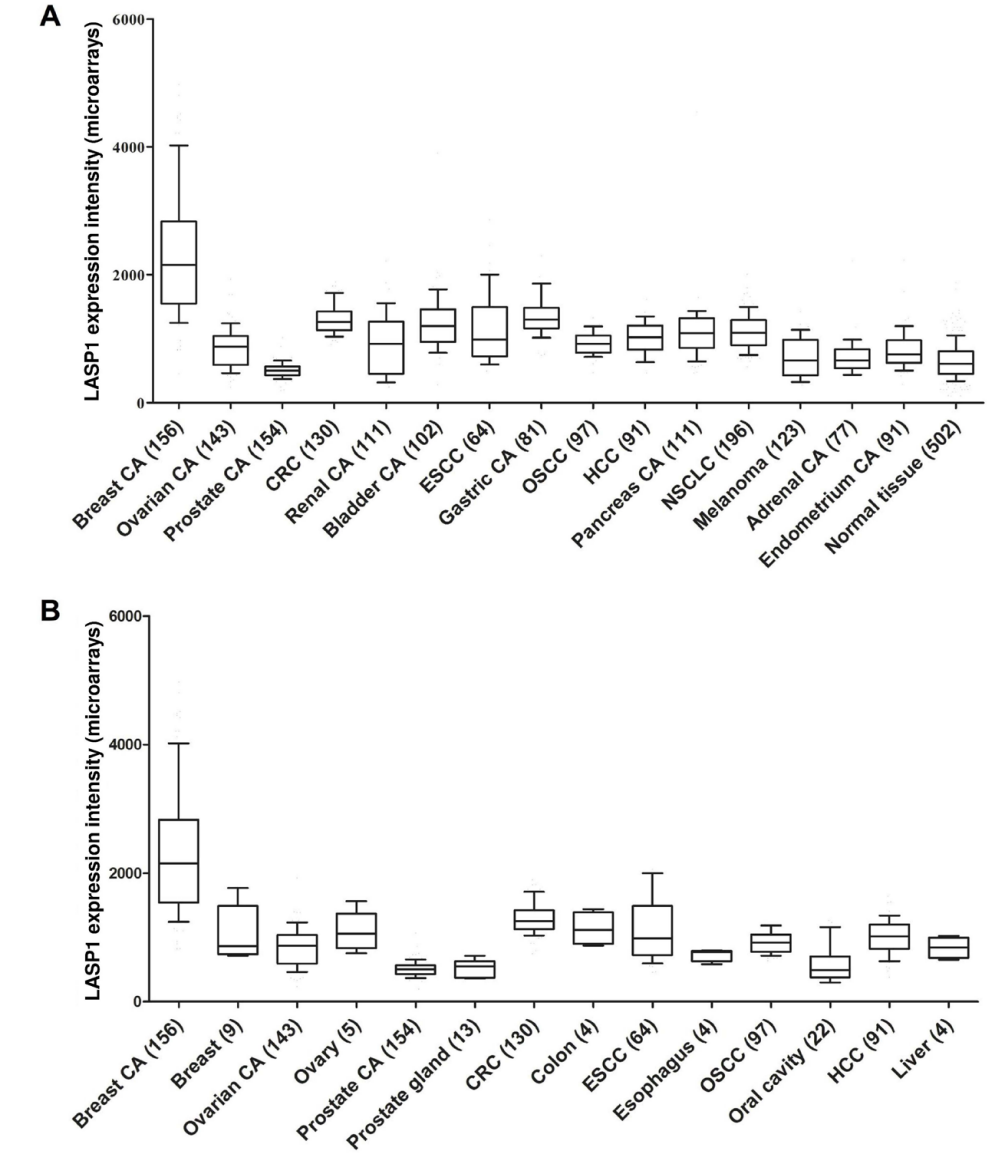
Gene expression patterns of LASP1 in human carcinoma tissues A) Gene expression patterns of LASP1 in different carcinomas (CA) compared to normal-body-atlas (normal tissue). B) Gene expression patterns of LASP1 in selected carcinomas in comparison with adjacent normal tissues. Publicly available microarray datasets (all Affymetrix HG-U133plus2.0) were retrieved from the GEO or the Array Express database at the EBI and normalized simultaneously by RMA using customized brainarray CDF files (ENTREZG v17) yielding one optimized probe-set for each transcript [106,107]. Accession codes: Breast CA n=156 GSE50948, ovarian CA n=143 GSE12172 GSE18520, prostate CA n=154 GSE17951, CRC n=130 GSE37892, renal CA n=111 GSE11151 GSE19982 GSE36895, bladder CA n=102 GSE31684 GSE7476, ESCC n=64 GSE26886 GSE32701 GSE42363, gastric CA n=81 GSE13911 GSE22377, OSCC n=97 GSE41613, HCC n=91 GSE9843, pancreas CA n=111 GSE17891 GSE32688 GSE42952 E-MEXP-2780, non-small-cell lung carcinoma (NSCLC) CA n=196 GSE37745, melanoma n=123 GSE35640 GSE15605, adrenal CA n=77 GSE10927 GSE19750, endometrium CA n=91 GSE17025, normal tissue n=502 GSE7307. All data shown as box plots. Whiskers indicate the 10^th^ and 90^th^ percentile. The number of samples is given in parentheses.

LASP1 is significantly overexpressed in numerous different cancer entities (Fig. 3) and affects tumor aggressiveness.

### 6.1. LASP1 overexpression in breast carcinoma

Concerning LASP1, the best-studied cancer entity to date is breast carcinoma. Initially, LASP1 was reported to be overexpressed in 8% of human breast carcinomas and expression was correlated to gene amplification based on data from BT-474 breast cancer cell line and primary tumors [1,2,59]. At that time, prostate derived ETS factor (PDEF), a potential tumor suppressor, was discussed as a regulator of LASP1 expression. PDEF inhibits cell migration in multiple invasive breast cancer cells, and transfection of PDEF in three invasive breast cancer cell lines resulted in regulation of proteins involved in cytoskeleton regulation, including LASP1 [60]. However, PCR analyses of laser-capture microdissected breast cancer cells as well as immunohistological analyses of a large series of breast cancers by Frietsch *et al*. demonstrated that LASP1 overexpression is neither mediated by copy number gains nor correlated with PDEF and p53 expression [61]. LASP1 overexpression in breast cancer was confirmed by immunohistochemistry (IHC), using an immunoreactivity score, a semi-quantitative rating for cell staining: The score was significantly higher in invasive breast carcinomas compared to mammary fibroadenomas. Moreover, the observed high cytoplasmic expression of LASP1 in breast cancer tissue correlated with a hitherto unknown nuclear LASP1-positivity [62].

Additionally, a co-overexpression of LASP1 with the ERBB2 (Her2/neu) oncogene was reported for human breast cancer tissues, as both genes are in close vicinity on the 17q11-21 region. This region also harbors the gene encoding for the tumor suppressor BRCA1, that is altered in 20-30% of breast cancers and most often accompanied by an amplification of *ERBB2* [2]. In this respect, also a correlation between LASP1 levels and ERBB2 expression was observed [63], however, a later study could not confirm this result [62]. As LASP1 is also upregulated in lactating mammary tissue [64], a possible regulation by prolactin can be assumed.

Recently, LASP1 was identified as a fusion partner of the zinc-finger transcription factor TRPS1 in the breast cancer cell line ZR-75-30 [65]. The oncogenic potential of this TRPS1-LASP1 fusion protein remains to be determined.

### 6.2. LASP1 overexpression in other human carcinomas

Immunohistochemical staining of cancerous ovarian tissue detected a strong expression of LASP1 [66]. LASP1 overexpression is associated with mucinous histology in primary tumors and was identified using array comparative genomic hybridization to be likely caused by gains of the 17q12-q24 locus [67].

Increased LASP1 protein levels were also found in metastatic high-risk prostate carcinomas [68], metastatic colorectal carcinomas (CRC) [69,70], in clear cell renal cell carcinoma (ccRCC) tissues [71] and in bladder carcinoma (BC) specimens [72]. Moreover LASP1 overexpression is observed in oral squamous cell carcinoma (OSCC) [73], esophageal squamous cell carcinoma (ESCC) [74], gastric cancer [75] and pancreatic ductal adenocarcinoma (PDAC) [76].

Additionally, in hepatocellular carcinoma (HCC) LASP1 expression, cytoplasmic as well as nuclear, was increased compared to adjacent non-tumoral tissue and was correlated with hepatitis B surface antigen and serum alpha-fetoprotein level of HCC patients [77], suggesting a link between LASP1 and tumorigenesis upon viral hepatitis. Consistently, LASP1 expression and relocalization from the cytoplasm to pseudopods is increased in the HCC HepG2 cell line by the hepatitis B virus X protein (HBx), the causing agent for HCC development by Hepatitis B virus infection. This influence on the subcellular localization is also observed in the HCC Huh-7 cell line, where LASP1 was mainly localized in the perinuclear fractions upon HBx expression. Upregulation of LASP1 by HBx is PI3-kinase (PI3K) pathway-dependent [78]. In HCC, LASP1 is further regulated by urokinase plasminogen activator (uPA), whose expression is an unfavorable prognostic factor and a therapeutic target in HCC: LASP1 is downregulated upon uPA inhibition and conversely uPA upregulation increases LASP1 expression and affects cell motility. In return LASP1 knockdown increases via disruption of actin microfilaments uPA secretion and cell motility [79].

Similarly to breast cancer and HCC, a nuclear LASP1 localization and overexpression has been confirmed for ESCC, mainly by confocal microscopy, immunohistochemistry, and Western blots of cytoplasmic and nuclear preparations [62,74,77]. These data, together with the fact that nuclear LASP1 correlates with worse prognosis [61], suggests a relevant effect of nuclear LASP1 on tumorigenesis and tumor progression.

Our comprehensive analysis of publicly available gene expression microarray data confirmed a high significance (p<0.001) for LASP1 mRNA overexpression in human carcinomas compared to adjacent normal tissues for breast carcinoma, ESCC and OSCC (Fig. 3B).

### 6.3. Functional role of LASP1 in human carcinoma

Tumor-derived cell lines have frequently been used for functional analysis of LASP1 expression in human carcinoma. In these models LASP1 expression was regulated by transient knockdown with LASP1 specific siRNA or overexpression.

In breast cancer cell lines BT-20 and MCF-7 siRNA-mediated LASP1 knockdown led to decreased cell migration and proliferation. Consistently, LASP1 overexpression in the non-cancer PTK-2 cell line, which does not express endogenous LASP1, resulted in a significant increase in cell motility [22]. Similar results concerning impaired proliferation and migration upon LASP1 knockdown were obtained with SKOV-3 ovarian cancer cell line [66].

Likewise, RNA interference with LASP1 reduced migration and proliferation of LNCaP prostate cancer cells [68] and SW620 CRC cells [69]. Conversely, ectopic LASP1 overexpression in SW480 CRC cells, which minimally express LASP1 under normal conditions, resulted in an aggressive phenotype with promoted tumor growth and metastasis in a murine xenograft model [69].

To explore the molecular mechanism of LASP1 on tumor progression, Wang *et al.* analyzed in SW480 and HCT116 cell lines the effect of LASP1 on proteins involved in the epithelial-mesenchymal transition (EMT), a process by which epithelial cells lose their cell polarity and cell-cell adhesion and gain migratory and invasive properties. Overexpression of LASP1 in CRC correlated inversely with the epithelial markers E-cadherin and ß-catenin while vimentin, a mesenchymal marker, was enhanced. Furthermore, LASP1 induced phosphorylation of proteins involved in the MAPK, PI3K/Akt and Smad signaling pathways and upregulated S100A4, a cytosolic and nuclear protein known to be involved in the regulation of cellular processes such as cell cycle progression and differentiation. Accordingly, LASP1 and S100A4 expression was upregulated upon TGFβ signaling and required for TGFβ-mediated EMT [70]. As EMT is an important step in tumor progression and metastasis, these data provide for the first time a possible mechanism of a LASP1-mediated effect on cancer aggressiveness.

Further studies of other tumor entities give evidence for LASP1 mediated cancer aggressiveness as silencing of LASP1 in ECA109 and KYSE510 ESCC cell lines significantly inhibited cell proliferation, migration and invasion *in vitro* [74]. For gastric cancer, the same has been shown *in vitro* using SGC7901/shLASP1 cells and was confirmed in SCID mice as tumorigenesis and metastasis were inhibited upon LASP1 knockdown [75]. For PDAC, reduced cell migration and invasion upon LASP1 knockdown have been shown in two cell lines with high endogenous LASP1, CFPAC-1 and MIA-PaCa-2, while overexpression in the BxPC-3 and Panc-1 cell lines with low endogenous LASP1 increases cell migration and invasion [76]. Moreover in HCC, LASP1 enhanced cell proliferation and migration, thus leading to more aggressive cancer cell phenotypes [77,78], while LASP1 knockdown in HBx-expressing HepG2 and Huh-7 cells significantly suppressed hepatocellular cell proliferation and migration [78]. In addition, upon LASP1 silencing in OSCC HSC-3 and Ca9-22 cell lines a significant inhibition of proliferation was observed *in vitro* and *in vivo* [73]. More, in ccRCC 786-0 cells RNA interference-mediated LASP1 silencing significantly inhibited cell migration [71]. Studies of BC cell lines treated with LASP1 specific siRNA again demonstrated significant cell viability, migration and invasion inhibition after LASP1 knockdown [72].

Interestingly, nuclear LASP1 localization has a peak in the G2/M phase during proliferation [61] and LASP1 silencing reduces proliferation by cell cycle arrest in the G2/M phase, as shown for BT-20 breast cancer cell line [22], ovarian cancer SKOV-3 cell line [80] and OSCC cell lines [73], indicating a role of LASP1 in cell cycle progression.

Collectively, these observations suggest that LASP1 overexpression in multiple cancers is not a mere passenger event, but rather a driver of tumorigenesis and cancer progression.

Of note, *Lasp1* knockout mice were reported to exhibit increased rates of wound healing and higher incidence of chemically induced skin tumors with greater average number of tumors. Besides, murine embryonic fibroblasts from *Lasp1* knockout mice showed faster migration rates, possessed increased focal adhesion numbers and displayed higher attachment rates. However, the LASP1-binding and focal adhesion protein LPP is overexpressed about twofold in these mice and might, in part, compensate for LASP1 loss in cytoskeleton assembly. Thus, there is a striking contrast in the functional effects between murine *Lasp1* knockout primary cells and human cancer cell lines [81].

### 6.4. LASP1 in medulloblastoma and leukemia

Besides studies on LASP1 in carcinomas, LASP1 was also investigated in leukemia and in medulloblastomas, the latter being one of the most common malignant pediatric brain tumors. Treatment failure mainly occurs in children affected by metastases from high-risk medulloblastomas, which are characterized by aberrations of chromosome 17, mostly deletion of 17p and gain of 17q with *LASP1* being one of the most upregulated genes on chromosome 17q in tumors with 17q gain. LASP1 knockdown experiments in medulloblastoma cell lines DAOY, UW228-2, and D283 showed a strong reduction of cell migration and proliferation, and an increased cell adhesion [82] pointing to an oncogenic role of LASP1 in medulloblastoma.

**Figure 4:**
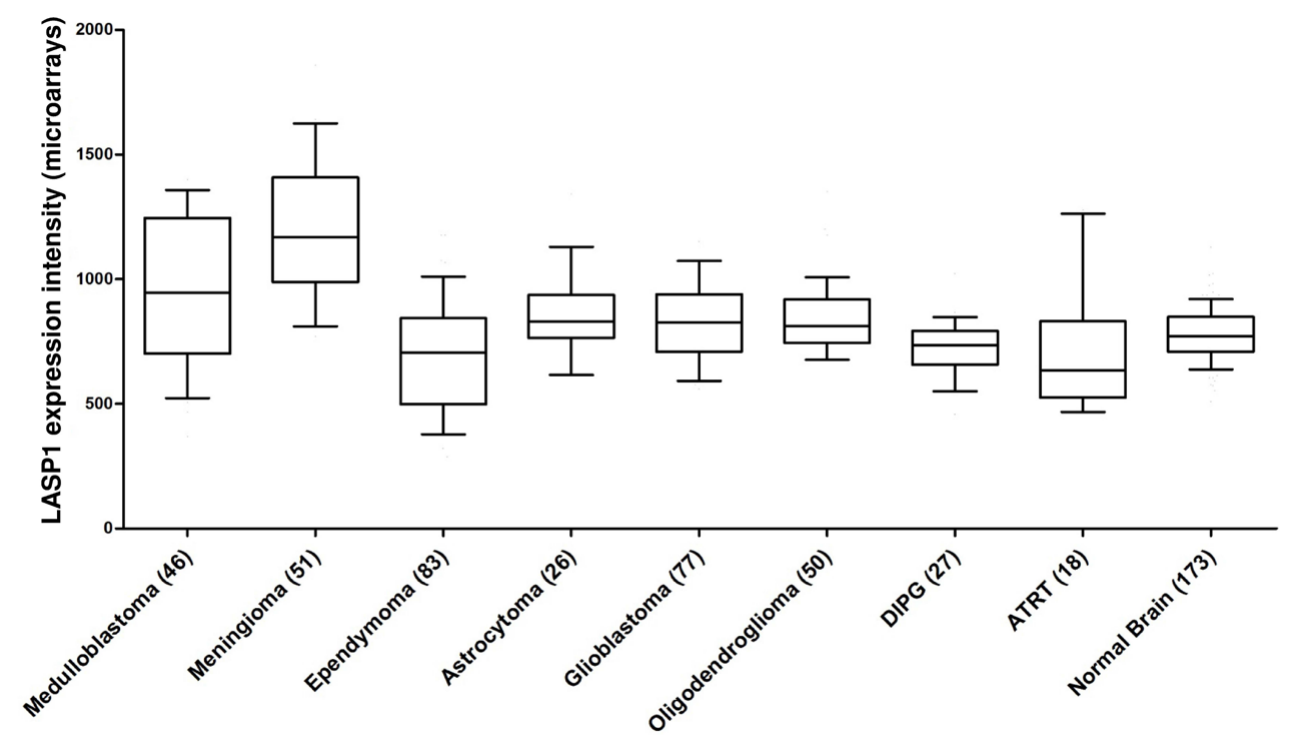
Gene expression patterns of LASP1 in neuronal tumor tissues: LASP1 is overexpressed highly significantly in medulloblastoma and meningioma as compared to normal brain tissues Publicly available microarray datasets (all Affymetrix HG-U133plus2.0) were retrieved from the GEO and normalized simultaneously by RMA using customized brainarray CDF files (ENTREZG v17) yielding one optimized probe-set for each transcript [106,107]. Accession codes: Medulloblastoma n=46 GSE10327, meningioma n=51 GSE4780, ependymoma n=83 GSE21687, astrocytoma n=26 GSE4290, glioblastoma n=77 GSE4290, oligodendroglioma n=50 GSE4290, diffuse intrinsic pontine glioma (DIPG) n=27 GSE26576, atypical teratoid/rhabdoid tumor (ATRT) n=18 GSE28026, normal brain n=173 GSE11882. Data are represented as box plots. Whiskers indicate the 10^th^ and 90^th^ percentile. The number of samples is given in parentheses.

Additionally in meningioma, an effect of LASP1 overexpression can be assumed as our comprehensive expression analysis revealed a highly significant (p<0.001) overexpression of LASP1 in this entity as well as in medulloblastoma. For glioblastoma and oligodendroglioma a significant LASP1 overexpression (p<0.05 and p=0.01, respectively) was observed. In contrast, expression analysis for ependymoma and diffuse intrinsic pontine glioma (DIPG) exhibited significant LASP1 underexpression (p<0.005 and p<0.05, respectively) as compared to normal brain tissues (Fig. 4).

There is growing evidence for a role of LASP1 in leukemia. *LASP1* was identified to be a fusion partner for *MLL*, a gene that is frequently rearranged in acute myeloid leukemia. The *MLL-LASP1* fusion gene is a result of a chromosomal translocation involving chromosomal segments 11q23 and 17q12-25. This translocation generates five distinct fusion genes. The MLL-LASP1 fusion was observed in only one out of 14 cases [83]. Both, the MLL-LASP1 and the reciprocal LASP1-MLL fusion protein enhance proliferation in mouse embryonic fibroblasts (MEFs), also in cotransfected cells. However, the effect of MLL-LASP1 is anchorage-dependent, while LASP1-MLL alone or cotransfected with MLL-LASP1 could stimulate proliferation in both, anchorage-dependent and -independent manner. Additionally, only the LASP1-MLL transfected and cotransfected MEFs were capable to form foci *in vitro* giving first evidence for oncogenic behavior [84].

In contrast to its expression in other leukemias, LASP1 is overexpressed and a direct substrate of the permanently active BCR-ABL oncogene in chronic myeloid leukemia (CML). Hyper-phosphorylation of LASP1 and CRKL, one of the most prominent and specific BCR-ABL substrates and a biomarker in CML, by BCR-ABL results in a disrupted interaction between both proteins, while normal binding occurs between tyrosine 171 phosphorylated LASP1 and the SH2 domain of non-phosphorylated CRKL [85]. The functional significance of this disturbed protein-protein interaction with respect to CML progression is still under investigation.

## 7. LASP1 expression regulation in human cancer

As overexpression of LASP1 is frequent in human cancer and associated with tumor aggressiveness, numerous studies investigated the regulation of LASP1 expression and identified several microRNAs (miRNAs), that target the 3′ untranslated region (UTR) of LASP1 [72,86]:

In CRC cell lines, LASP1 was identified as a direct target of miR-133a and downregulation of miR-133a was observed in 85% of primary tumors and in 100% of liver metastases [86,87]. Ectopic miR-133a expression impaired cell proliferation and migration, and sufficiently suppressed tumor growth and metastasis in liver and lung *in vivo* [86], indicating that miR-133a can act as tumor suppressor. In contrast, the subgroup of CRC patients with higher, but still downregulated miR-133a expression developed more often distant metastases, presented advanced Dukes and TNM staging and showed poor survival [88]. Notably miR-133a expression not only reduces the expression of LASP1 but also of key cellular molecules like Rho GDI 1, Rab GDI-β and proteins involved in the MAPK pathway, hence inhibiting phosphorylation of ERK and MEK [86]. Similar results on tumor suppressive behavior were obtained for another LASP1 targeting miRNA in CRC: miR-1. Exogenous miR-1 inhibited MAPK as well as PI3K/Akt signaling and further increased epithelial markers, thus reversing EMT. These effects are most likely mediated by targeting LASP1 as LASP1 increases MAPK signaling and drives EMT [86,89]. Finally in prostate cancer, LASP1 regulation at its 3′ UTR by miR-1 was demonstrated [90].

Another prominent LASP1 regulating miRNA is the tumor suppressor miR-203 [91]. MiR-203 is often silenced in different malignancies, such as ESCC [92] and prostate cancer [68,93], and its expression levels are inversely correlated with those of LASP1 in ESCC [92] and prostate cancer [68,91]. In human head and neck squamous cell carcinoma, genetic reconstitution experiments proofed the direct effect of miR-203 on LASP1 (and on SPARC and NUAK1) and suppressed pro-metastatic cell activities [94].

A significant downregulation of miR-203 is also reported in triple-negative breast cancer cell lines. Upregulation of miR-203 expression inhibited cell migration - similar to siRNA-mediated LASP1 knockdown. Conversely, up-regulation of LASP1 abrogated the effects induced by miR-203 transfection in these cell lines [95]. These data indicate that the effect of miR-203 on cell function is mainly mediated by LASP1 downregulation.

Parallel studies discussed miR-218 to be involved in LASP1 overexpression, as miR-218 is downregulated in prostate cancer [96]. In bladder carcinoma, luciferase reporter assays showed reduced luminescence intensity with miR-1, miR-133a, and miR-288 transfectants, suggesting cognate target sites in the 3′UTR of LASP1 for these miRNAs [72].

Besides regulation by miRNAs, LASP1 is a bona fide repressed target of the tumor suppressor p53 [97], as seen by functional repression effect of p53 on LASP1 via p53-response element in HCC. Hence, p53 mutations at key residues involved in DNA binding abrogated the p53-mediated suppression of LASP1 expression [98]. As p53 is inactivated by somatic mutations in about 50% of human cancers, the loss of p53 activity may account for the lion's share of LASP1 overexpression observed. On the other hand, not all tumors with a defect p53 tumor suppressor show increased LASP1 protein levels [61].

In pancreatic ductal adenocarcinoma (PDAC) *LASP1* has been identified as a target of the hypoxia regulated transcription factor HIF-1α. Under hypoxia HIF-1α binds specifically to one of four hypoxia response elements in the promoter region of human *LASP1*, hence increasing its expression. Therefore, LASP1 expression correlates with the HIF-1α expression in PDAC cell lines as well as in human specimens of PDAC [76].

## 8. LASP1 as cancer biomarker

Overexpression of LASP1 is associated with increased tumor aggressiveness of numerous cancers [61,68,71,75-77,82,99] (Table 2), suggesting that LASP1 protein levels may serve as a prognostic marker.

**Table 2:** Prognostic relevance of LASP1 expression in human cancer Statistical significance is indicated by asterisks (*p.0.05; **p.0.01; ***p.0.005; ****p.0.001). ccRCC: clear cell renal cell carcinoma; DFS: disease free survival; HCC: hepatocellular carcinoma; IHC: immunohistochemistry; OS: overall survival; PDAC: pancreatic ductal adenocarcinoma; PFS: progression free survival; RFS: recurrence free survival.

Tumor entity	LASP1 expression correlates with	Cohort size	Detection method	References
Breast carcinoma	- DFS **	20	SQ-PCR	[101]
	- ki67 positivity (for nuclear LASP1) *	177	IHC	[61]
	- reduced OS (for nuclear LASP1) *			
	- low-grade tumors (for nuclear LASP1) *			
Colorectal carcinoma	- clinical stage **	126	IHC	[69]
	- lymph node metastasis ***			
	- reduced OS ***			
Gastric cancer	- tumor size ****	126	IHC	[75]
	- invasive depth ***			
	- TNM stage *			
	- lymph node metastasis ****			
	- reduced OS *			
	- low p53 expression ****			
HCC	- HBsAg positivity (for cytosolic LASP1) *	144	IHC	[77]
	- reduced OS (for cytosolic LASP1) ***			
	- serum AFP level (for cytosolic/nuclear LASP1) ****/*			
	- patient's survival as an idependent marker (for cytosolic LASP1 in multivariate analysis) **			
Medullo-blastoma	- 17q gain ****/****	207/101	IHC/qRT-PCR	[82]
	- increased metastasis at diagnosis ****/***			
	- reduced OS ****/*			
	- reduced PFS ****	207	IHC	
PDAC	- TNM stage *	91	IHC	[76]
	- lymph node metastasis **			
	- reduced OS **			
Prostate carcinoma	- PSA progress (for cytosolic/nuclear LASP1) */*	193	IHC	[68]
ccRCC	- increased tumor size ***	216	IHC	[71]
	- TNM stage ***			
	- recurrence status **			
	- death status *			
	- reduced OS ***			
	- reduced RFS ****			

In fact, in gastric cancer and HCC multivariate analyses revealed, that cytoplasmic LASP1 expression is an independent prognostic factor of patients’ survival [75,77]. In other cancer entities LASP1 is a promising marker as part of a marker set, like in prostate cancer. There LASP1 is one protein in a set of 22 markers constituting a genomic classifier (GC). Within Gleason score groups, cases with high GC scores experienced earlier death from prostate cancer and reduced overall survival [100]. In breast carcinoma, a prognostic index was implemented, too. This index could predict the postoperative outcome with five genes including *LASP1*. Surprisingly these genes were underexpressed in 10 patients, that died within 5 years after surgery, in comparison with 10 patients with unaltered prognostic signature, who survived disease-free for more than 5 years [101]. However, these data need to be validated in a larger cohort to draw conclusions for individualized therapy.

Additionally, LASP1 was recently identified as one protein out of a set of potential markers to discriminate the differentiation status and metastatic behavior in CRC [99]. Zhao *et al*. showed that besides S100A9 and RhoGDI, detection of LASP1 by IHC predicts clinicopathological characteristics of CRC and that LASP1 expression increases from normal mucosa to CRC and metastatic CRC. Therefore the pattern changes of these proteins have the potential to be used for the design of marker panels for assistance in diagnostic and therapeutic strategies in CRC [102]. Moreover, LASP1 was identified as component of a six-gene signature, that is strongly predictive for disease progression and relapse in CML patients [103].

So far, these data indicate a lag in the potential of LASP1 to become a clinical parameter. Most expression data can only be collected by tumor extirpation and, hence, can only be used as a post-surgery prognostic marker, but not as a pre-surgery prognostic marker or even as a diagnostic marker. An exception is BC. Although LASP1 showed strong expression throughout the urothelium of the bladder and ureter, a modest overexpression in transitional cell carcinoma (TCC) specimens, the most common type of BC, was observed and the authors depicted LASP1 to be a sensitive biomarker for the presence of TCC in urinary cell pellets (sensitivity, specificity, positive and negative predictive values were determined to be 83.1%, 85.3%, 83.1% and 80.6%, respectively). However, urine contamination with erythrocytes above 250 cells/μl and urinary tract infections yielded false positive results, thus limiting the broad usability of urinary LASP1 levels as TCC-biomarker to cases without urinary tract infection or gross hematuria [104].

The possible benefit of LASP1 as a biomarker is not restricted to cancer, as LASP1 is one out of 12 differentially expressed proteins that show decreased expression in cultured skin fibroblasts of insulin-dependent type 1 diabetes mellitus patients with diabetic nephropathy versus diabetics without nephropathy or healthy subjects. Therefore, LASP1 might serve as prognostic marker for the development of diabetic nephropathy [105].

## 9. LASP1 is a nucleo-cytoplasmic shuttling protein

**Figure 5:**
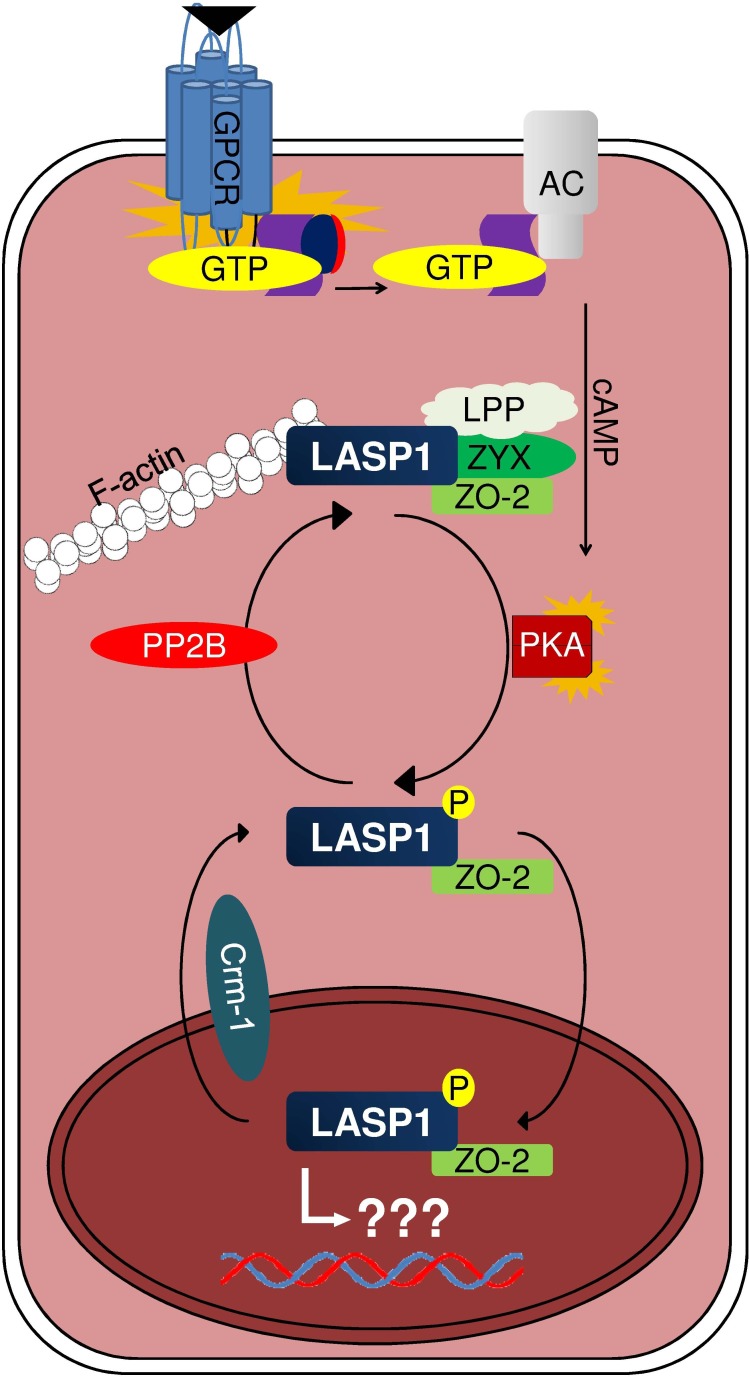
Proposed mechanism of LASP1 translocation AC: adenylyl cyclase; GPCR: G-protein coupled receptor; LPP: LIM domain containing preferred translocation partner in lipoma; PKA: cAMP-dependent protein kinase; PP2B: protein phosphatase 2B; ZO-2: zonula occludens protein 2; ZYX: zyxin.

A nuclear accumulation of LASP1 is observed in different cancer entities [62,68,74,77,82] and is associated with worse patient outcome in breast cancer [61]. Due to the lack of a nuclear import signal, LASP1 nuclear translocation requires binding to the shuttle partner zonula occludens protein 2 (ZO-2). Under basal conditions, LASP1 is localized at the cell membrane and to focal contacts by interacting with actin, zyxin, LPP and ZO-2. Phosphorylation of LASP1 by PKA at serine 146 abrogates binding to actin, zyxin and LPP and induces translocation of the unaltered LASP1/ZO-2 complex from the cytoplasm into the nucleus. Nuclear export is mediated by a newly identified conserved nuclear export signal between residues 71 and 77 (NLRLKQQ) in LASP1 (Fig. 1). Moreover, dephosphorylation of LASP1 by PP2B is suggested to relocalize the protein back to focal contacts (Fig. 5). Therefore LASP1 is a nucleo-cytoplasmic shuttling protein, transducing PKA-mediated signals into the nucleus [20]. The role of LASP1 inside the nucleus remains to be elucidated.

## CONCLUSION AND PERSPECTIVES

Two decades after the identification of LASP1, there is a still growing number of publications underscoring LASP1 as a truly versatile protein. Besides its role as a physiological structural, functional, and signaling protein, its clinical relevance as a prognostic marker in different cancers or even as a diagnostic marker in TCC emerges. So far, effects of LASP1 have been studied mostly in epithelial carcinomas while its role in other cancer entities like sarcomas remains unknown. Further investigations are warranted to implement LASP1 as a prognostic marker in routine diagnostics, possibly in combination with several biomarkers that can predict tumor aggressiveness, and would help optimizing individual therapy regimes.

While the basics of LASP1 protein regulation e.g. by miRNAs and p53, becomes better understood, there is a lag of knowledge about the precise molecular mechanism of how LASP1 acts on cell migration, proliferation, and cell cycle control, possibly in interaction with its numerous binding partners or via conveying extracellular signals from the membrane into the nucleus. Although LASP1 is a proven nucleo-cytoplasmic shuttling protein, its functional role in the nucleus remains largely elusive. Therefore, future studies will have to dissect the molecular function of LASP1, especially in the nucleus, and to validate its contribution to cancer progression and its value as a prognostic or even diagnostic biomarker.

## ABBREVIATIONS

AC: adenylyl cyclase
ATRT: atypical teratoid/rhabdoid tumor
BC: bladder cancer
CA: carcinoma
ccRCC: clear cell renal cell carcinoma
CXCR: chemokine receptor
CML: chronic myeloid leukemia
CRC: colorectal carcinoma
DFS: disease free survival
DIPG: diffuse intrinsic pontine glioma
EMT: epithelial-mesenchymal transition
ESCC: esophageal squamous cell carcinoma
F-actin: filamentous actin
Ft: fetal tissue
GC: genomic classifier
GEO: Gene Expression Omnibus
GPCR: G-protein coupled receptor
HBx: hepatitis B virus X protein
HCA: homocysteic acid
HCC: hepatocellular carcinoma
IHC: immunohistochemistry
Krp1: kelch related protein 1
LASP1: LIM and SH3 protein 1
LPP: LIM domain containing preferred translocation partner in lipoma
MEF: mouse embryonic fibroblast
miRNA: microRNA
NES: nuclear export signal
NSCLC: non-small-cell lung carcinoma
OS: overall survival
OSCC: oral squamous cell carcinoma
PDAC: pancreatic ductal adenocarcinoma
PDEF: prostate derived ETS factor
PFS: progression free survival
PI3K: PI3-kinase
PKA: cAMP-dependent protein kinase
PKG: cGMP-dependent protein kinase
PP2B: protein phosphatase 2B
RMA: Robust Multi-array Average
RFS: recurrence free survival
SH3: SRC homology region 3
SNP: single nucleotide polymorphism
TCC: transitional cell cancer
uPA: urokinase plasminogen activator
UTR: untranslated region
VASP: vasodilator-stimulated phosphoprotein
ZO-2: zonula occludens protein 2
ZYX: zyxin
